# Opisthobranch grazing results in mobilisation of spherulous cells and re-allocation of secondary metabolites in the sponge *Aplysina aerophoba*

**DOI:** 10.1038/s41598-020-78667-7

**Published:** 2020-12-14

**Authors:** Yu-Chen Wu, María García-Altares, Berta Pintó, Marta Ribes, Ute Hentschel, Lucía Pita

**Affiliations:** 1 Marine Ecology, GEOMAR Helmholtz Centre for Ocean Research, Düsternbrooker Weg 20, 24105 Kiel, Germany; 2 Christian-Albrechts University of Kiel, Düsternbrooker Weg 20, 24105 Kiel, Germany; 3 Department Biomolecular Chemistry, Leibniz Institute for Natural Product Research and Infection Biology-Hans Knöll Institute (HKI), Adolf-Reichwein-Straße 23, 07745 Jena, Germany; 4 Department of Animal Biology, University of Barcelona, Barcelona, Catalonia Spain; 5 Department Marine Biology and Oceanography, Institute of Marine Sciences (ICM-CSIC), Passeig Marítim de la Barceloneta, 37-49, 08003 Barcelona, Catalonia Spain

**Keywords:** Chemical ecology, Marine biology, Marine chemistry

## Abstract

Sponges thrive in marine benthic communities due to their specific and diverse chemical arsenal against predators and competitors. Yet, some animals specifically overcome these defences and use sponges as food and home. Most research on sponge chemical ecology has characterised crude extracts and investigated defences against generalist predators like fish. Consequently, we know little about chemical dynamics in the tissue and responses to specialist grazers. Here, we studied the response of the sponge *Aplysina aerophoba* to grazing by the opisthobranch *Tylodina perversa,* in comparison to mechanical damage, at the cellular (via microscopy) and chemical level (via matrix-assisted laser desorption/ionization imaging mass spectrometry, MALDI-imaging MS). We characterised the distribution of two major brominated alkaloids in *A. aerophoba*, aerophobin-2 and aeroplysinin-1, and identified a generalised wounding response that was similar in both wounding treatments: (i) brominated compound-carrying cells (spherulous cells) accumulated at the wound and (ii) secondary metabolites reallocated to the sponge surface. Upon mechanical damage, the wound turned dark due to oxidised compounds, causing *T. perversa* deterrence. During grazing, *T. perversa*’s way of feeding prevented oxidation. Thus, the sponge has not evolved a specific response to this specialist predator, but rather relies on rapid regeneration and flexible allocation of constitutive defences.

## Introduction

Many sessile marine organisms have developed chemical defences to avoid predators, compete for space, and prevent fouling and colonisation by pathogens and opportunistic microbes. These defences may be constitutive (always present in the organisms), inducible upon stimuli (synthesised anew), or activated when the organism is attacked (converted from a non- or less toxic precursor to a potent toxin)^1–3^. Nevertheless, some specialised predators have overcome these chemical defences in order to take advantage of an otherwise unexploited food source. These specialists are often small animals (mesograzers) for which their prey also becomes their habitat since, despite its poor nutritional value, feeding on chemically-defended organisms provides the additional benefit of protection^1,4,5^. Thus, chemical defences mediate species-species interactions and determine organisms’ success, shaping the diversity and function of benthic communities^6^.

Sponges represent a prominent example of chemically-defended marine organisms. They have been extensively studied because of the potential medical and biotechnological application of their secondary metabolites and constitute the richest source of marine natural products^7–10^. Each sponge species produces a specific, yet diverse chemical arsenal with fish-deterrent, antifouling and antimicrobial properties, to name a few^3,4,11–13^. Despite these chemical defences, a great variety of beautiful opisthobranchs are specialised in living and feeding on one or a narrow range of sponge species. These specialised sea slugs defend themselves by accumulating and modifying the secondary metabolites they acquire from the sponges they eat^4^. Despite these well-known associations, and the extensive literature on sponge chemical defences, we know little about the response of sponges to grazing by these specialists and whether it differs from the response to predation by generalists or wounding. Moreover, most research has focused on the concentration and bioactivity of secondary metabolites in crude extracts and we lack spatial resolution on the distribution of compounds within individual sponges.

Here, we investigated the interaction between the sponge *Aplysina aerophoba* (Nardo, 1833) and the sea slug *Tylodina perversa* (Gmelin, 1791). Sponges of the genus *Aplysina* serve as models for sponge chemical ecology^14–19^ because they are widely distributed and present similar chemical profiles, dominated by brominated isoxazoline alkaloids^20,21^. This genus has stimulated a heated debate on activated defences in sponges (e.g.,^22–24^). In *A. aerophoba*, in vitro assays showed that the most abundant isooxazoline alkaloids (aerophobin-2, isofistularin-3, and aplysinamisin-1) constituted the precursors of the smaller, more cytotoxic compounds aeroplysinin-1 and its structurally related dienone^13,22,23,25^. The precursors showed repellence activity against marine fishes^26^, but the bioconversion products displayed enhanced toxicity and antimicrobial activity^19,23^, hinting at an activated defence based on rapid transformation of the chemical precursors into the more bioactive forms upon cell disruption^22,23,25^. This transformation was proposed to be enzymatically-mediated^22,25^, but no enzyme involved in the bioconversion of precursors to aeroplysinin-1 or dienone has been identified so far. The studies suggesting enzymatic bioconversion and activated defences in *Aplysina* sponges have been highly criticised, mainly because of possible methodological artefacts (e.g. working with freeze-dried sponges) and because experiments on two Caribbean *Aplysina* species did not support activated antipredatory defences^24^. A latter study addressing those critics^23^ argued that the absence of activated defence in the Caribbean *Aplysina* species reported in^24^ lies on the correlation between bioconversion and wound intensity^23^. These works exemplify that it remains unclear to what extent and under which conditions sponges may show flexible antipredatory defences.

Despite being heavily chemically-defended, *A. aerophoba* is grazed by the sea slug *T. perversa.* While feeding, this sea slug selectively sequesters and accumulates sponge-derived brominated alkaloids, in particular aerophobin-2^27^. It also accumulates *A. aerophoba*’s saffron-coloured pigment uranidine, which makes *T. perversa* camouflage with the sponge^28^. We performed controlled experiments to characterise the response of *A. aerophoba* to grazing by *T. perversa*. To elucidate if the sponge response is specific to grazing by this specialist and/or requires the predator presence, we included a treatment in which sponges were mechanically-damaged by clipping with a scalpel. At the cellular level, we focused on the fate of spherulous cells, in which brominated compounds are stored^29^, via light and transmission electron microscopy. At the chemical level, we visualised the distribution of two of the main brominated compounds in *A. aerophoba*, aerophobin-2 (precursor) and aeroplysinin-1 (bioconversion product)^23,25^ by Matrix-Assisted Laser Desorption-Ionization imaging Mass Spectrometry (MALDI-imaging MS), in order to avoid bias derived from chemical extraction procedures. Under the hypothesis of bioconversion, we expected to detect higher concentrations of aeroplysinin-1 in wounded than in control samples, and the opposite pattern for aerophobin-2. We further tested whether the chemical and cellular responses upon mechanical damage translated into increased deterrence capability against *T. perversa*.

## Methods and materials

### Animal collection

**Figure 1:**
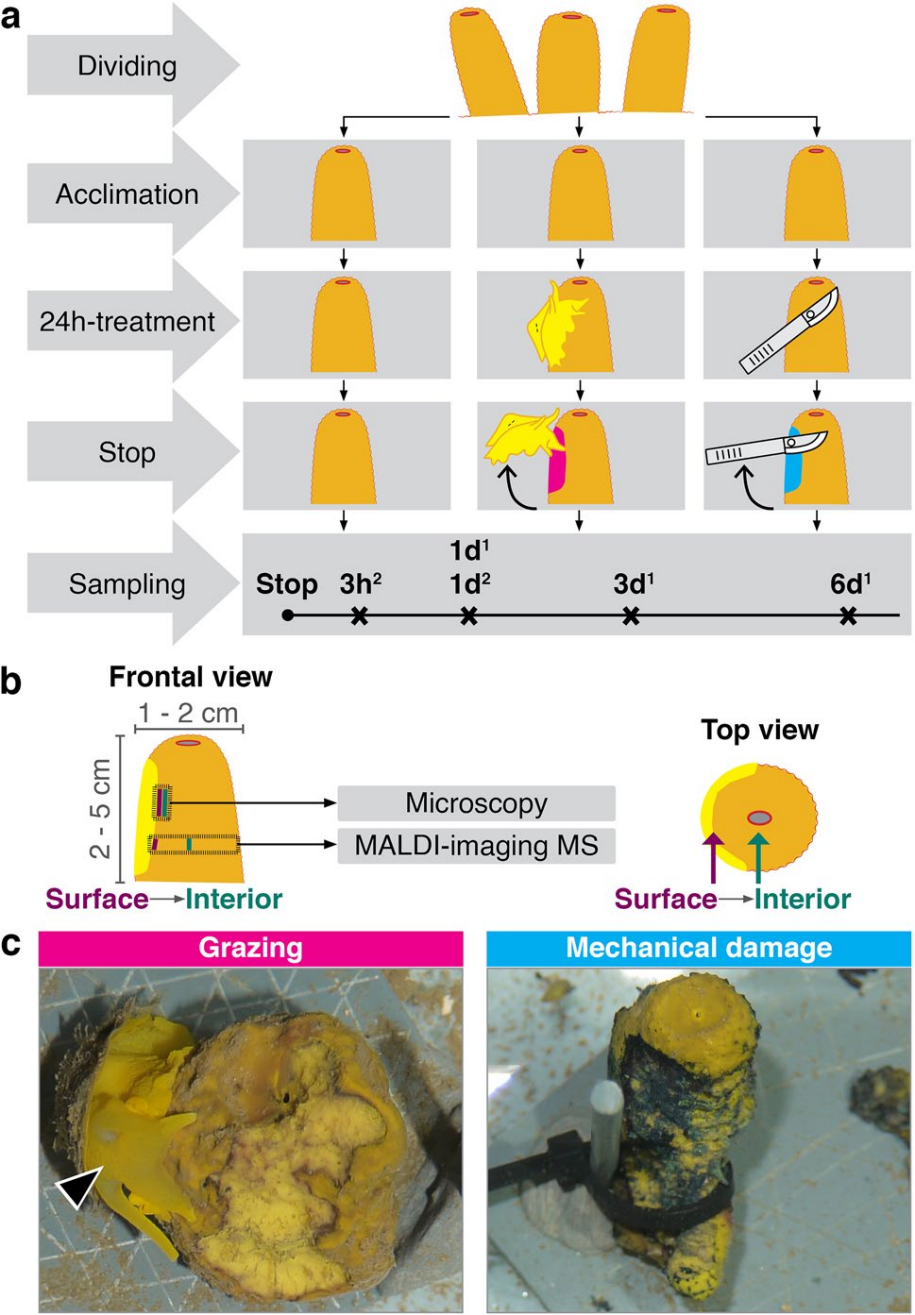
Experimental design. (**a**) Each sponge individual was divided into three specimens that were randomly assigned to either control (left panels), grazing (middle panels), or mechanical damage (right panels) treatment. Treatment was applied for 24 h. We performed consecutive experiments to collect samples at different time points: 3 h, 1 day, 3 days, and 6 days post-treatment. Superscript number 1 and 2 denotes experiments performed in 2016 and 2017, respectively. (**b**) Size of the sponges and sampling area for microscopy and MALDI-imaging MS. The corresponding surface (purple) and the interior (dark green) of sponge samples and the wound (yellow) are also shown. Histological cross-sections of the sponge tissues (a view from the top) were collected for MALDI-imaging MS analysis. (**c**) Wound by *T. perversa* (arrowhead, left panel) and by mechanical damage (right panel). The figure layout and figure panels (**a,b)** were generated in Scribus (version 1.5.5., URL: http://www.scribus.net).

The sponge *Aplysina aerophoba* and the sea slug *Tylodina perversa* were collected by Scuba diving in June 2016 and May 2017 at the Mediterranean coast of Spain (42.29408 N, 3.28944 E and 42.1145863 N, 3.168486 E, respectively), at a depth between 2 to 10 m. Animals were immediately transported to the Experimental Aquaria Zone at the Institute of Marine Science (ICM-CSIC) in Barcelona (Spain). There, each sponge individual was divided into 2–3 specimens, each with a single osculum, and attached to plates by cable ties (Fig. 1). Each specimen was placed into individual 6L aquaria and maintained in a flow-through system with direct intake of seawater.

### Experimental set-up

After 1 week acclimation, the specimens from each sponge individual were randomly assigned to one of the following treatments: (i) control—no treatment; (ii) grazing—one sea slug, that had been starved for 24 h, was placed in direct contact to the sponge specimen and allowed to feed ad libitum for 24 h; and (iii) mechanical damage—the sponge specimen was clipped at the surface with a scalpel for 3 min every half hour for the first 3 h and the last 3 h of the 24 h-treatment period (Fig. 1a). All treatments were stopped after 24 h. Each experiment had the same experimental design but differed in the sampling point post-treatment (Fig. 1a): three experiments were performed in 2016, in which samples were collected 1 day, 3 days, and 6 days post-treatment; two experiments were performed in 2017 and samples were collected 3 h and 1 day post-treatment (*n* = 3–4 for all experiments, Supplementary Table 1 online). No mechanical damage group was performed in the 3-days experiment. The sampling areas of sponge tissue for microscopy and MALDI-imaging MS is shown in Fig. 1b.

### Sample preparation for light microscopy

Samples were immediately fixed in 2.5% glutaraldehyde in 0.1 M cacodylate buffer/11% sucrose and stored at 4 ºC. Samples were then processed as in^30^. Shortly, samples were washed with cacodylate buffer and post-fixed in 2% osmium tetroxide, followed by a dehydration in a graded ethanol series. After gradual infiltration with LR White resin, samples were polymerised in this resin. Semi-thin (0.35 μm) sections were prepared with an ultramicrotome (Reichert Ultracut S, Leica, Austria), deposited on Superfrost Plus glass slides (Menzel) with Biomount 2 mounting medium (BBI Solutions), stained with Richardson solution, and imaged (100× magnification) with a ZEISS Axio Observer microscope (version 1.1, Zeiss, Germany).

### Automatic counting of spherulous cells

Microscopic images were analysed in ImageJ (version 1.51j8, Java 1.8.0_112)^31^. The first 100 μm from the surface to the interior was defined as the Region Of Interest 1 (ROI 1, Supplementary Fig. 1 online) and its area was measured excluding aquiferous canals. Image type, Subtract background, Threshold, and Watershed parameters were adjusted to select densely-stained spherulous cells. Next, those cells were automatically counted by using ImageJ tool “Analyse Particles” with cell size from 34 to 314 μm^2^ and cell circularity from 0.20 to 1.00 (considering the variable size and shapes of spherulous cells^32,33^). Spherulous cells on edges of each ROI were not counted. The number of densely-stained spherulous cells per 50,000 μm^2^ in ROI 1 (100 µm × 500 µm) was calculated for each image (Supplementary Fig. 1 online) and compared amongst treatments by applying Generalised Linear Mixed-effects Model via Penalised Quasi-Likelihood (glmmPQL, MASS package^34^) in R (v3.6.0)^35^ as implemented in RStudio (v1.2.1335)^36^, with treatment as the fixed effect and sponge individual as the random effect. The distribution pattern of the densely-stained spherulous cells from the surface to the interior of sponges was investigated by defining five consecutive 100 µm-deep ROIs adjacent to ROI 1 (Supplementary Fig. 1 online) and counting cells as well as performing the statistical comparison of each ROI amongst treatments following the methodology described above. The automatic counting was validated by manual counting on a subset of samples (Supplementary Fig. 1 online).

### Transmission electron microscopy (TEM)

One sponge individual from 1 day, with its corresponding three differently-treated specimens, was analysed by TEM in order to characterise the spherulous cells observed by light microscopy. Embedded samples were cut into ultra-thin (70 nm) sections using an ultramicrotome (Reichert Ultracut S, Leica, Austria). Sections were mounted on pioloform coated grids and contrasted with uranyl acetate replacement stain (Science Services, Germany) for 20 min and subsequently with Reynold´s lead citrate for 3 min. Ultra-thin sections were imaged with a Tecnai G2 Spirit BioTwin transmission electron microscope (80 kV, FEI, USA) at the Central Microscopy of University of Kiel (Germany).

### Sample preparation for MALDI-imaging MS

The spatial distribution of secondary metabolites within sponge cross-sections was assessed by MALDI-imaging MS. Sponge tissue samples from 1 day-2017 experiment (*n* = 4 individuals × 3 treatments) were wrapped in aluminium foil (with a clear annotation of the wound location in grazing and mechanical damage treatments), snap-frozen in liquid nitrogen, and immediately stored at − 20 °C. Samples were prepared as described by Yarnold et al*.*^37^, with some modifications (see details in Supplementary Information online). In short, each sample was cryo-sectioned at 14 μm in a cryostat (CM3050 S, Leica, Germany) and mounted onto Indium-Tin-Oxide (ITO, Bruker Daltonics, Bremen, Germany) glass slides. Each ITO glass slide consisted of three sections corresponding to three sponge specimens (control, grazed, and mechanically-damaged) from the same sponge individual. Unlike Yarnold et al.^37^, the sample sections were directly mounted onto the slides without previous washing with MilliQ-water, as this step caused a morphological alteration of our sections resulting in the delocalisation of the compounds of interest. After light microscopy imaging (100× magnification), each ITO glass slide was coated with universal MALDI matrix chosen for optimal visualisation of both aerophobin-2 and aeroplysinin-1, which differ in polarity. Further information on the optimization of the sample preparation process can be found in^30^.

### MALDI-imaging MS analysis

In MALDI-imaging MS, a laser is passed over the sample and, due to the matrix, compound ions are released and passed to the mass analyser. At each raster point, mass spectrometry data is obtained and then integrated into an image in a specialised software. Here, raster widths of 250, 275, and 300 μm were selected according to the section area and in order to keep analysis time below 5–6 h and, therefore, preserve sensitivity and consistency during measurement (Supplementary Table 2 online). Samples were analysed in an UltrafleXtreme MALDI TOF/TOF (Bruker Daltonics), operated in positive reflector (detailed analysis is provided in Supplementary Information online).

Brominated compounds with two bromine atoms (Br) show a specific three-peak-pattern because the two stable isotopes (^79^Br and ^81^Br) occur in similar abundance in nature (ratio 50.5:49.5). Thus, aerophobin-2 and aeroplysinin-1 were identified by their molecular mass and isotopic pattern. Commercially available aerophobin-2 and aeroplysinin-1 (Santa Cruz Biotechnology, Germany) served as standard references to investigate ionization yields at the same concentration (1 mM). Since aerophobin-2 and aeroplysinin-1 showed different ionization yields, relative intensity of each compound between treatments was qualitatively compared. First, MALDI-imaging MS datasets were Root Mean Square normalised. Then, the intensity for each compound was shown as relative intensity to the highest value among the three sections within each ITO glass slide (i.e., among the specimens of the same individual), and depicted in a colour scale.

We investigated the co-localisation of aerophobin-2 and aeroplysinin-1. Ion images were exported as greyscale images in SCiLS Lab 2016b (Bruker; a software for analysis of mass spectrometry imaging data) so that in each pixel, the absolute intensity of each compound was computed as relative values of a gradient grayscale (0 to 255). The resulting images were imported in Fiji^38^ and analysed as follows: Co-localisation images show the pixels where both compounds had a value > 0 in the greyscale (abbreviated AND). Occurrence images show the pixels where at least one of the two compounds had a value > 0 in the greyscale (abbreviated OR).

MALDI-imaging MS allows the untargeted monitorisation of *A. aerophoba* metabolites, with thousands of complex spectra per sample. Compounds containing Br were manually annotated according to their isotopic profile. Moreover, the whole datasets were explored by a multivariate statistical tool, spatial segmentation: Spectra were clustered by their similarity, generating an unsupervised spatial segmentation map of each tissue section where regions of similar chemical compositions (i.e. clusters) were revealed and depicted with a distinct colour^39^. Segmentation maps were calculated in SCiLS Lab 2015b by bisecting k-means using correlation distance, weak denoising and Root Mean Square normalisation (peak picking workflow: 200 peaks (every 2 spectra).

### Deterrence experiments

We tested if *T. perversa* preferred control over mechanically-damaged sponges in deterrence experiments. In the 2017 animal collection effort, we took one chimney of five individuals of *A. aerophoba* and five individuals of *T. perversa*. We ran the experiment 1 day after collection and prepared the experimental setup following suggestions in^40^. For this experiment we did not work with sponge specimens but with sponge pieces: One hour before the experiment, each sponge chimney was divided into apical, middle, and bottom portions. Each portion was separated through the middle of the osculum (ca. 3 to 6 cm^3^) into two pieces which were randomly assigned to either control or mechanical damage treatment. Mechanical damage was applied during 5 min by clipping the surface with a scalpel and the wound was 1 cm^2^ and 1–2 mm deep. No clipping was performed on control pieces. To test deterrence, we covered *T. perversa* with an opaque vessel for 15 min. We placed two pieces of the same sponge individual and region, one mechanically-damaged and one control, at 5 cm of the sea slug and we removed the vessel. We considered that the sea slug made a choice when it touched the sponge with the oral tentacles or with the front head within the first 15 min after removal of the vessel (Supplementary video 2 online). Experiments with different sea slug individuals and portions of the sponge (apical/middle/bottom) were performed in separate aquaria in the open flow-through system. *T. perversa* preference was tested in a Binomial test (p = q = 0.5) in R (v3.6.0)^35^ as implemented in RStudio (v1.2.1335)^36^.

## Results

Once the experiment started, each sea slug usually took 15–30 min to start feeding on the sponge (Supplementary video 1 online). They tightly attached and covered the sponge with the mantle during grazing. The oscula of control group sponges were usually wide open, whereas they were less open or even closed in grazing or mechanically-damaged group sponges. Sea slugs usually remained at the same spot on the sponge during the 24 h feeding period, turning over their bodies to feed neighbouring tissue. The wounds generated by grazing showed a bright yellow colour, similar to the ones observed in the field, whereas the wounds in mechanical damage were dark blue (Fig. 1c). When grazing-induced wounds showed some darkening, it was comparatively minor and occurred at the frontier between the wound and the intact tissue.

### Accumulation of spherulous cells at the wound

**Figure 2:**
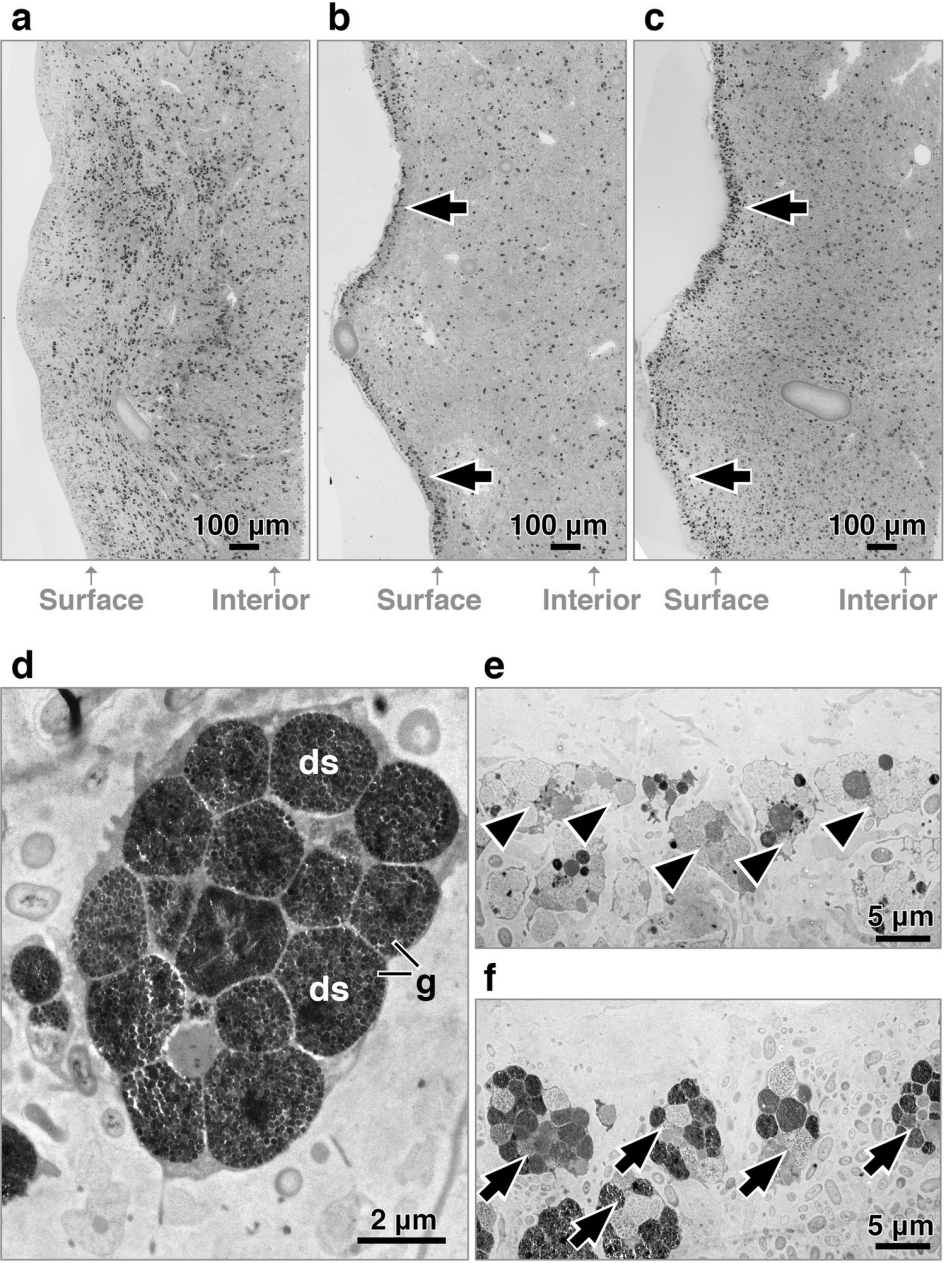
Accumulation of spherulous cells with electron-dense spherules at the surface 1 day post-treatment. Microscopic section (100× magnification) of 1d-samples showing that, compared to control **(a)**, densely-stained spherulous cells accumulated at the wound (arrow) in grazed **(b)** and mechanical damage **(c)** groups. (**d**) TEM-image at the wound confirming that densely-stained spherulous cells correspond with spherulous cells containing electron-dense spherules (ds), with numerous electron-dense granules **(g)**. (**e**) The surface of control group contained spherulous cells with electron-lucent spherules (arrowhead). (**f**) The surface of wounding group contained spherulous cells with electron-dense spherules (arrow).

Images (100× magnification) of specimens collected 1 day post-treatment showed a striking accumulation of densely-stained cells at the injured surface (first 100 μm from the wound to the sponge interior) in both grazed and mechanically-damaged specimens but not in the controls (Fig. 2). Further inspection by TEM confirmed that those cells were spherulous cells with electron-dense spherules (Fig. 2). In contrast, the surface of the control group contained mostly spherulous cells with electron-lucent spherules (Fig. 2). In addition, shedding of spherulous cells and the presence of cell debris at the wound site and the aquiferous canals occurred more frequently in both grazed and mechanically-damaged specimens than in the control (Supplementary Fig. 2 online).

**Figure 3:**
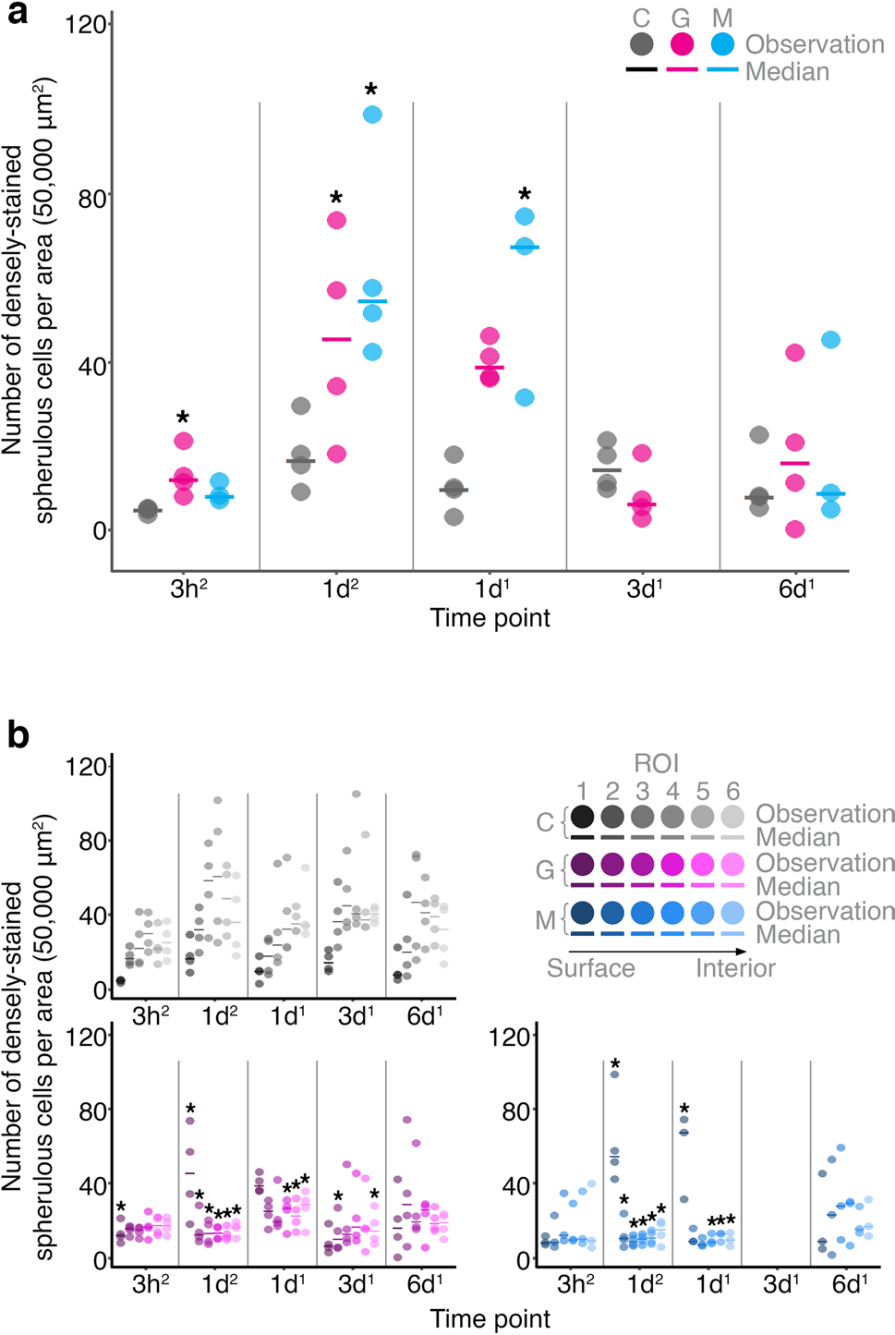
Time-dependent re-distribution of spherulous cells upon wounding. Number of densely-stained spherulous cells per area (50,000 μm^2^) right at the surface (first 100 μm) (**a**) and within the first 600 µm (**b**). Spherulous cell density in grazing and mechanical damage were compared to the control at each Region Of Interest (ROI, each defined by a depth of 100 µm, starting from the surface, ROI 1, consecutively to the interior ROI 6). Note that there was no mechanical damage group at 3 days. Superscript number 1 and 2 denotes experiments performed in 2016 and 2017, respectively. Star indicates statistically significant differences. *C* control; *G* grazing; *M* mechanical damage.

We further quantified the accumulation of densely-stained spherulous cells (termed spherulous cells for the following text) at different time points. Upon grazing, spherulous cells gathered at the surface 3 h after treatment (glmmPQL, *p* = 0.023). After 1 day, the density of spherulous cells at the surface reached the highest value in both the grazed and mechanically-damaged groups (Fig. 3a) and represented a significant accumulation compared to control samples, except in 1 day-2016 grazing group (glmmPQL; 1 day-2016: grazing, *p* = 0.056 and mechanical damage, *p* = 0.022; 1 day-2017: grazing, *p* = 0.035 and mechanical damage, *p* = 0.013). In contrast, 3-days and 6-days samples showed a similar density of spherulous cells at the surface amongst all treatments (glmmPQL, *p* > 0.1).

We also investigated the distribution of spherulous cells from the surface to 600 µm interior of the tissue. In the control group, the density of spherulous cells followed a depth-dependent distribution pattern with a lower value at the surface (first 100 µm) compared to the inner tissue, with the highest value at a depth of ca. 300–400 μm (Fig. 3b). After grazing and mechanical damage, this distribution pattern was disrupted (Fig. 3b). At 3 h after wounding, sponges displayed a similar density of spherulous cells from the wounded surface to the interior of the sponge specimens. At 1 day, the cell density significantly peaked at the wounded surface (ROI 1, first 100 µm) compared to the surface in control (Fig. 3a), whereas for the other ROIs (i.e., towards the sponge interior), the number of spherulous cells was similar or significantly lower than in the control (Fig. 3b, Supplementary Table 3). After 3 days, the distribution pattern of spherulous cells resembled that observed in control group (Fig. 3b, Supplementary Table 3), consistent with the initiation of regeneration (Supplementary Fig. 3 online). After 3 days, the ectosome showed a well-defined surface border with less densely-stained spherulous cells (Supplementary Fig. 3 online).

### High inter-individual variability of the distribution of brominated alkaloids but distinct allocation upon wounding

**Figure 4:**
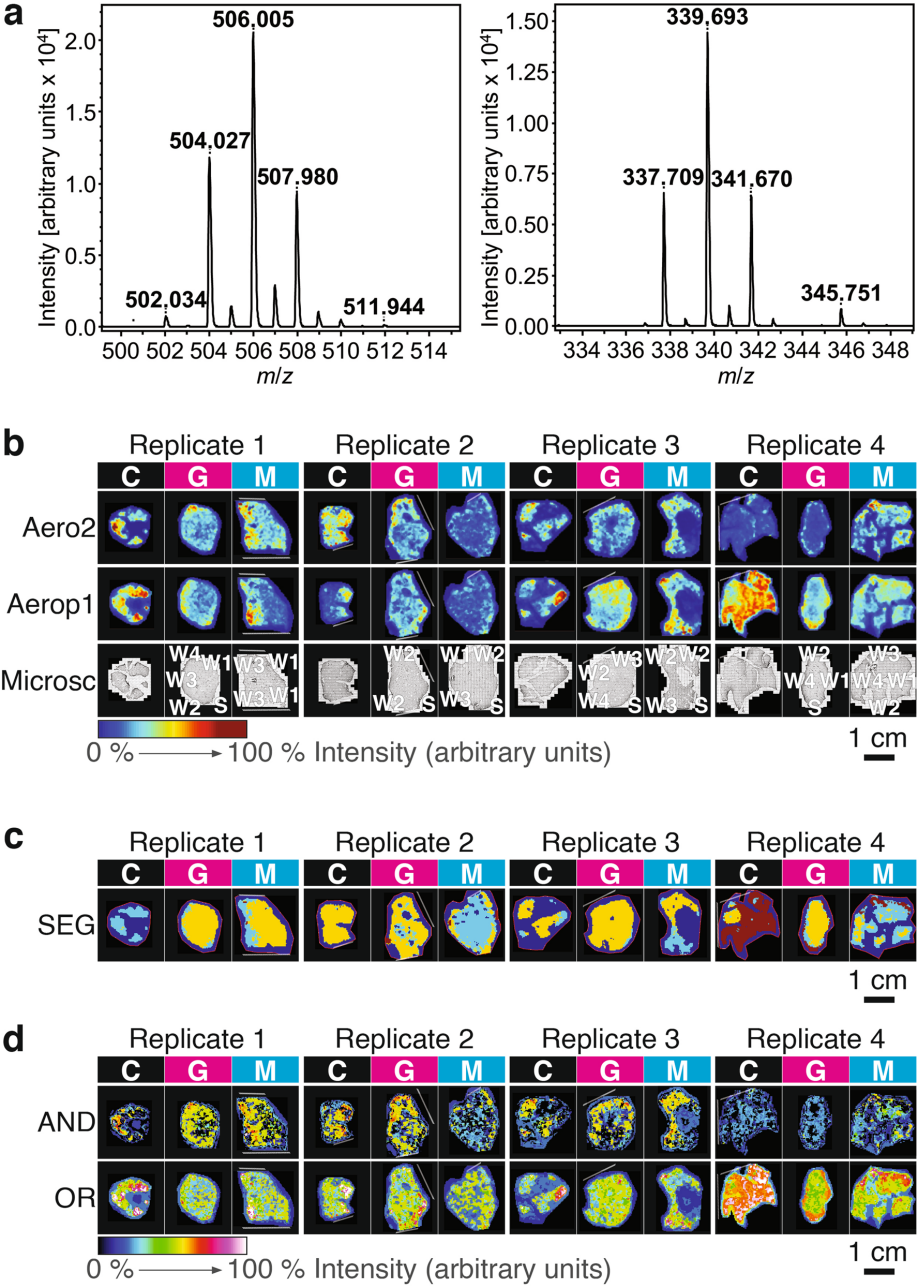
MALDI-imaging MS of sponges 1 day post-treatment. (**a**) The experimental isotopic patterns of aerophobin-2 (left panel) and aeroplysinin-1 (right panel) by MALDI-imaging MS. (**b**) The relative abundance of these compounds (*Aero2* aerophobin-2, *Aerop1* aeroplysinin-1) in the three different treatments (*C* control, *G* grazing, *M* mechanical damage) for each biological replicate (replicate; i.e., sponge individual). The corresponding microscopic images (Microsc) showing the location of the wounds are also shown, i.e. *S* surface (non-wounded); *W1* the first evident wound; *W2* the second evident wound, and so on. (**c**) Segmentation maps (SEG). Each cluster represents a chemical profile and is assigned a distinct colour that is comparable between treatments within each replicate. **(d)** Images of the co-localisation of aerophobin-2 and aeroplysinin-1 (AND), the distribution of at least one of these two compounds (OR, occurrence maps). The relative intensity from 0 to 100% is depicted in a colour scale with warmer colours representing relatively higher intensity and colder colours lower intensity of each compound. White-dotted line = broken or cut edges.

We visualised the abundance of two of the main brominated alkaloids (aerophobin-2 and aeroplysinin-1) on the cross-sections of *A. aerophoba* tissues of sponges within the 1-day after treatment by MALDI-imaging MS. Both aerophobin-2 and aeroplysinin-1 occurred in all samples (experimental monoisotopic *m/z* 504.0 corresponding to C_16_H_20_Br_2_N_5_O_4_ for aerophobin-2, and monoisotopic *m/z* 337.7 corresponding to C_9_H_10_Br_2_NO_3_ for aeroplysinin-1). These isotopic patterns agreed with their molecular formula and matched the isotopic patterns of the standards (Fig. 4a). MALDI-imaging MS revealed, in all samples, other compounds that are likely brominated alkaloids according to their isotopic patterns. The ones with higher abundance (at least 10 times more intense than the baseline) are shown in Supplementary Fig. 4 online. These Br-containing metabolites might be those already reported in *Aplysina* species, or related to known *Aplysina* brominated alkaloids. For instance, *m/z* 420.954 fits with Aplysamine-1 ([M + H]^+^ C_16_H_27_Br_2_N_2_O) and *m/z* 1136.347 may be related to fistularins (putative molecular formula [M + H]^+^ C_33_H_31_Br_6_N_4_O_11_)) (Supplementary Fig. 4 online).

MALDI-imaging MS revealed a striking biological variability of the distribution of both aerophobin-2 and aeroplysinin-1 among the four control samples (Fig. 4b) and their distribution did not follow a specific trend (i.e., the compounds were not preferentially found in either the interior or the surface). In contrast, the distribution patterns of aerophobin-2 and aeroplysinin-1 in treated individuals (G and M) was more consistent among biological replicates and apparently more similar to each other than to their respective controls (Fig. 4b). In treated individuals, aerophobin-2 and aeroplysinin 1 tended to be more abundant at the surface (Fig. 4b). Since most brominated compounds follow a similar trend (Supplementary Fig. 5 online), we suggest that both grazing and mechanical damage induced a re-distribution of brominated alkaloids.

Unsupervised segmentation maps based on the 200 most intense peaks were calculated to determine and visualise “chemical regions”, i.e. regions of the sample with distinct chemical composition^39^. These maps reflect groups of pixels with similar mass spectra (they contain similar peaks with similar intensities) grouped in color-coded clusters. In general, we observed that segmentation maps from control specimens only had one cluster (one colour), or one cluster covered most of the tissue, indicating that the chemical composition of control specimens is relatively homogeneous, although there is also variability among individuals (Fig. 4c). However, in most treated specimens, segmentations maps revealed a different clustering than the control, with mainly two clusters (two colours) correlating with the accumulation patterns of aerophobin-2 and aeroplysinin-1, which tended to present higher abundance at the surface of the sponge (Fig. 4b).

Using the distribution of aerophobin-2 and aeroplysinin-1, we calculated the co-localisation maps (Fig. 4d, AND, representing pixels where both compounds were present) and the occurrence maps (Fig. 4d, OR, representing pixels where at least one compound is present). These maps revealed two trends, regardless of the treatment: (i) co-localisation maps (Fig. 4d, AND) resembled the spatial distribution of aerophobin-2, suggesting that when aerophobin-2 is detected, aerooplysinin-1 is usually detected as well; (ii) areas with the highest intensity in the occurrence maps (Fig. 4d, OR) usually corresponded to no signals in the co-localisation maps, i.e. only on compound accounts for the signal in those areas. Since the location of these high intensity areas resembled the spatial distribution of aeroplysinin-1, we infer that where aeroplysinin-1 is very abundant, aerophobin-2 is not, which suggests interconversion.

### Correlation between brominated alkaloids and spherulous cell accumulation

**Figure 5:**
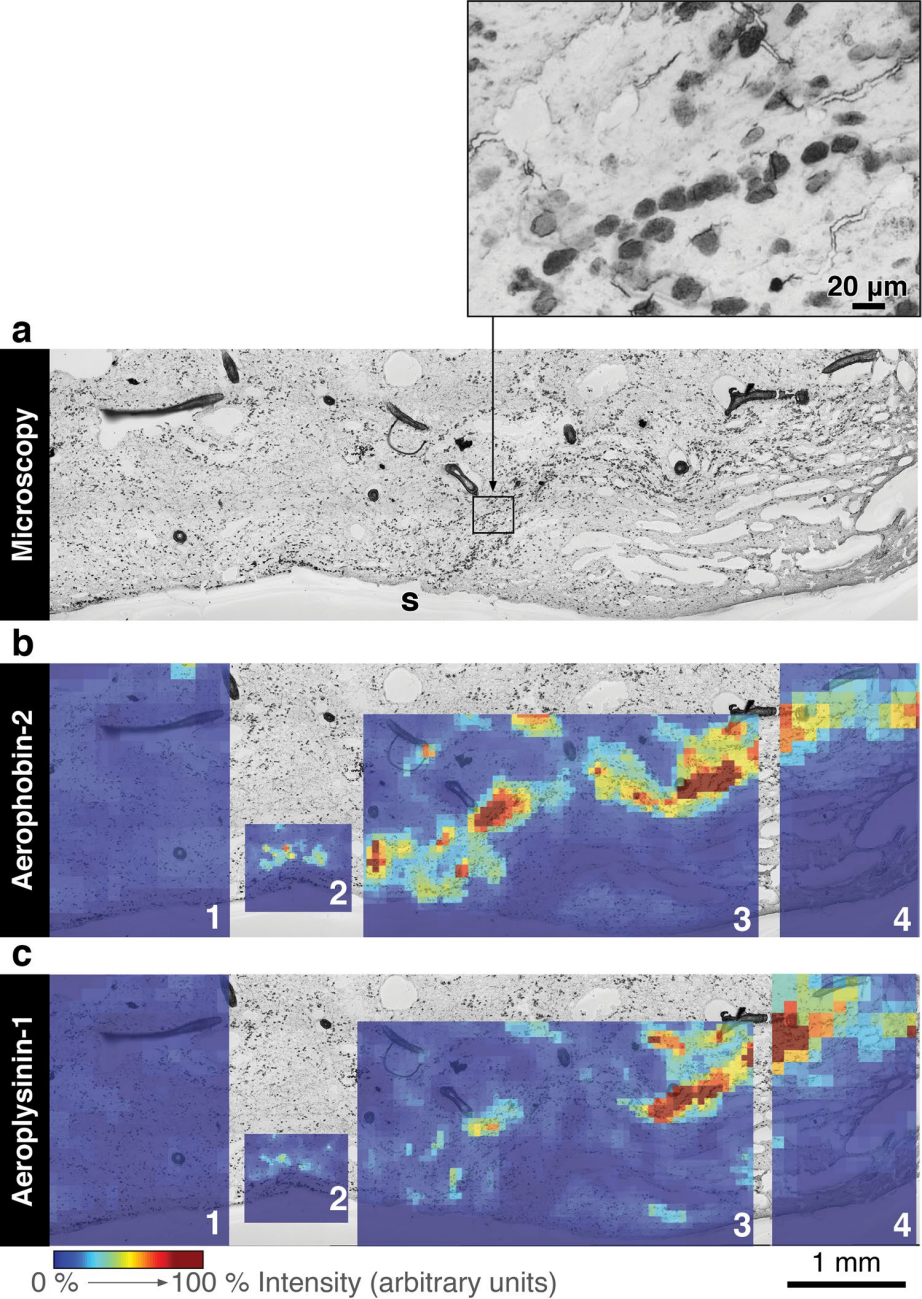
Correlation of cellular pattern and spatial distribution of brominated compounds. (**a**) Light microscopic image of a grazed sample collected from the field. A track of spherulous cells was parallel to the surface beneath the subdermal spaces below surface (s). The size, shape and location of the main cell type (a size from 10 to 20 μm, densely-packed spherule-like structures, location beneath subdermal spaces) resembled that of spherulous cells, as shown in the insert. Superimposition of the microscopic image with 2D-MALDI-images of aerophobin-2 (**b**) and aeroplysinin-1 (**c**), respectively. The sample was measured by MALDI-imaging MS with a raster size at 100 μm (1 and 4), 50 μm (3), and 20 μm (2). The relative intensity of each compound is depicted in a colour scale. The superimposition of microscopic images was generated in SCiLS Lab 2015b (Bruker; a software for analysis of mass spectrometry imaging data, URL: https://scils.de). The figure layout was processed in Scribus (version 1.5.5. URL: http://www.scribus.net).

The different resolution of the microscopy images used for automatic counting and the MALDI-imaging MS images prevents us from unequivocally assigning the accumulation of brominated alkaloids to the accumulation of spherulous cells at the wound. In MALDI-imaging MS technology, we ran the samples at a resolution of 250–300 μm to ensure the comparability of the within-tissue distribution of target compounds among the different treatments, while keeping the experiments short enough to maintain constant intensity (Fig. 4). However, we did analyse one grazed sponge collected from the field at higher spatial resolution (20–100 μm) and we observed that a “track” of spherulous cells co-localised with peaks of both aerophobin-2 and aeroplysinin-1 (Fig. 5).

### *Tylodina perversa* avoids mechanically-damaged sponges

In the deterrence experiments, if a choice was made (Supplementary Video 2 online), *T. perversa* always preferred control sponge pieces over mechanically-damaged ones (Table 1; Binomial test with p = q = 0.5, P = 0.002). Mechanically-damage sponge pieces were characterised by the dark blue colouration of the wound. One *T. perversa* individual remained still in all experiments and made no choice. This individual was bigger than the others and showed less mobility. The experiment included different portions of chimney to reduce potential intra-individual variability and we observed fewer choices in the experiments including the middle part of the sponge (Table 1).

**Table 1:** Feeding choice experiments in *Tylodina perversa*.

Feeding choice	N	No choice	Choice
Control: mechanical damage	15	6	9:0
Chimney portion—apical:middle:bottom			4:1:4
*T. perversa* individual—1:2:3:4:5			2:0:3:2:2
* aerophoba* individual—1:2:3:4:5			2:1:2:2:2

The total number of replicates is expressed by “N”. “No choice” denotes those experiments where *T. perversa* did not show a preference within 15 min after the sponges were presented. “Choice” shows the preference and number of times that option was selected.

## Discussion

Our results show that the response of *A. aerophoba* to grazing by the specialist *T. perversa* resembles the response to the mechanical damage treatment. MALDI-imaging MS showed that the brominated alkaloids aerophobin-2 and aeroplysinin-1 are constitutively present in all sponge samples and their distribution within the tissue varies among individuals. Yet, these secondary metabolites were consistently re-allocated to the surface upon wounding. Spherulous cells, carrying brominated alkaloids, gathered at the wound 1 day post-wounding. The different distribution of spherulous cells from the surface to the interior in treated vs control samples suggests that this accumulation results from an active migration of these specialized cells to the wound. After 3 days, coincident to visible signs of tissue restoration, all treatments showed similar spherulous cell distribution. The time of the cellular response to wounding is similar to that reported in other sponge species^41,42^.

We propose that the active migration of spherulous cells plays a role in defence and regeneration upon wounding. Previous studies^29^ and the MALDI-imaging MS results at high spatial resolution (Fig. 5) support their function as carriers of the brominated alkaloids. We observed the shedding of spherulous cells upon damage and their release into aquiferous canals. In the absence of wounding, spherulous cells are found in the exhalant channels in different sponge species and it was proposed as a mechanism for excretion of metabolic by-products^33,43–45^. In the sponge *Crambe crambe,* the release of spherulous cells carrying toxic compounds was stimulated by mechanical stress in a process called spherulisation^46^. Thus, spherulisation may be also occurring in *A. aerophoba* as a mechanism for releasing chemical defences upon damage. Recently, Ereskovsky et al.^47^ described the accumulation of spherulous cells at the wound, 1 day after applying a small excision, as part of the regenerative blastema in *Aplysina cavernicola*. Our study shows a similar cellular response in *A. aerophoba*, in both mechanical damage and grazing treatments, even if in our study wounding damage was stronger than in Ereskovsky et al.^47^. We propose that, in addition to defence, they could contribute to structure the new tissue, like in the formation of propagules in *Chondrosia reniformis*^48^. We also speculate that the isoxazoline alkaloids carried by the spherulous cells could potentially be reutilised for nutrition at the wound, provided that the sponges (or its symbionts) can degrade them into primary metabolites, as shown in plants^49^. Therefore, the mobilisation of spherulous cells may be a common process involved in sponge defence, while also provides structure and even energy for rapid regeneration.

Our results suggest that the bioconversion between the precursor aerophobin-2 and aeroplysinin-1 is constitutive and occurred in all samples, including control as well as wounding treatments. It is remarkable that other Br-compounds followed a similar distribution as aerophobin-2 and aeroplysin-1 (Fig. 4c, Supplementary Fig. 5 online). We did observe that Br-compounds were less abundant in treated samples than in controls. One explanation could be their transformation to the related dienone, which we could not detect in our MALDI-imaging MS protocol, likely because dienone signal overlaps with background matrix signals. Another explanation could be their release to the environment via spherulisation.

MALDI-imaging MS revealed the dynamic nature of the sponges’ chemical response to predation and wounding with unprecedented spatial resolution. We observed high variability in the distribution of chemical compounds in control specimens, in concordance with the natural variability in absolute concentrations observed in previous chemical studies of *A. aerophoba*^18,50,51^, and other sponge species (reviewed in^12^). Several studies explored the differential allocation of secondary metabolites in sponges, with concentrations usually higher in the inner than the outer parts of the sponges^12^. This pattern may reflect the antibacterial activity function of secondary metabolites^16,52^. The numerous aquiferous canals in the inner sponge tissue effectively expand the surface area of the sponge exposed to the external environment and Optimal Defence Theory predicts that defences should be allocated in those regions that are most valuable and/or at higher risk. However, our results did not show a clear differential allocation of brominated secondary metabolites to either the surface or the interior of the sponge but rather strong individual variability in control samples. This variability contrasted to the redistribution of compounds upon wounding, with a tendency to gather at the surface; a pattern which seems to arise from a reallocation of existing defences rather than de novo synthesis. Such reallocation would have the advantage of using available resources to enhance the protection of the area that has now been signalled as most vulnerable to damage, fitting the postulates of Optimal Defence Theory. Moreover, this mechanism would provide a flexible use of chemical defences to adapt to different challenges and may explain the recurrent intraspecific variability in sponges.

Despite the similarity in the response upon grazing and upon mechanical damage, we did detect one prominent difference between the two treatments. Upon mechanical damage, sponge wounds turned black due to the oxidation of uranidine, which likely happens during cell damage or exposure to the surrounding environment^28^. Our deterrence experiments suggest that this reaction deters *Tylodina.* In fact, wounds in the grazing treatment remained mainly yellow. *Tylodina* grazes with its radula in a way that the wound remains covered by its mantle and/or by the mucus liberated by the slug during feeding. This may serve to reduce cellular damage and exposure of the wound to the environment; thus, preventing the formation of more deterrent sponge compounds.

Grazing by the specialist sea slug *T. perversa* triggers a wound-like response in the sponge *A. aerophoba*. This response consisted of a local accumulation of spherulous cells, which are likely directed to enhance regeneration^47^ as well as defend the exposed wound against invading microbes and predators^23^. After one day, coinciding with the peak of spherulous cell accumulation, brominated secondary metabolites are re-allocated to the surface of the sponge. We propose that wounding cues signal the surface as the region at most risk and thus, induce reallocation. We observed the darkening of the sponge wound immediately upon mechanical damage, which is likely due to the release of oxidised compounds^28^. Interestingly, *Tylodina* prevents this from happening, probably because it covers the wound with its mantle while feeding.

In conclusion, contrary to reports in other organisms such as algae or dinoflagellates^53–55^, the sponge has evolved an un-specific response to the specialist grazer. This “jack-of-all-trades” defence strategy relies on the constitutive production of a diverse chemical arsenal and the mediation of specialised cells to link defence and regeneration processes. Future studies using RNA-seq are on-going to decipher the underlying molecular mechanisms behind this defence strategy.

## Supplementary Information

Supplementary Video 1.

Supplementary Information 1.

Supplementary Video 2.

## Data Availability

Images derived from light microscopy and MALDI-imaging MS and videos recorded during the experiments are available at PANGAEA, https://doi.org/10.1594/PANGAEA.907958
