# Supplementary material for: Opisthobranch grazing results in mobilisation of spherulous cells and re-allocation of secondary metabolites in the sponge Aplysina aerophoba
Source: Sci Rep. 2020 Dec 14;10:21934. doi: 10.1038/s41598-020-78667-7 (PMC7736331; doi:10.1038/s41598-020-78667-7)
Supplement: Supplementary file 2 — Supplementary Information 1. [file 41598_2020_78667_MOESM2_ESM.pdf]

Supplementary Information

Opisthobranch grazing results in mobilisation of spherulous cells and re-allocation of secondary metabolites in the sponge *Aplysina aerophoba*

Yu-Chen Wu[1,2], María García-Altares[3] 0000-0003-4255-1487, Berta Pintó[4], Marta Ribes[5] 0000-0001-9747-295X, Ute Hentschel[1,2] 0000-0003-0596-790X, Lucía Pita[1*] 0000-0003-0163-1587

[1]GEOMAR Helmholtz Centre for Ocean Research, Marine Ecology, Düsternbrooker Weg 20, D-24105 Kiel, Germany

[2]Christian-Albrechts University of Kiel, Düsternbrooker Weg 20, D-24105 Kiel, Germany

[3]Leibniz Institute for Natural Product Research and Infection Biology – Hans Knöll Institute (HKI), Department Biomolecular Chemistry, Adolf-Reichwein-Straße 23, D-07745 Jena, Germany

[4]University of Barcelona, Department of Animal Biology, Barcelona, Spain

[5]Institute of Marine Sciences (ICM-CSIC), Department Marine Biology and Oceanography, Passeig Marítim de la Barceloneta, 37-49, E-08003 Barcelona, Catalonia, Spain

*****corresponding author: lpita@geomar.de

Protocol for MALDI-imaging MS sample preparation and analysis. Samples were cryosectioned as described by Yarnold *et al.* (2012), with some modifications. In short, each sample was thawed at RT and placed in a cryomold (22 mm in diameter, 5 mm in height, Tissue-Tek@ Cryomold@, Plano, Germany) with a drop of embedding medium, Optimal Cutting Temperature (OCT, Tissue-Tek@, Plano, Germany). If samples were bigger than the cryomold, excess sponge tissue was cut to fit the cryomold, preserving the surface. After filling with OCT, each sample was cryo-sectioned at 14 µm in a cryostat (CM3050 S, Leica, Germany) at -20 °C and then thaw-mounted onto Indium-Tin-Oxide (ITO, Bruker Daltonics, Bremen, Germany) glass slides for subsequent MALDI-imaging MS. Each ITO glass slide was spray-coated with 2 ml of a saturated solution (20 mg/mL) of universal MALDI matrix (1:1 mixture of 2,5-dihydroxybenzoic acid and α-cyano-4-hydroxy-cinnamic acid; Bruker Daltonics, Germany) in acetonitrile/methanol (70:30, v/v), using the automatic system ImagePrep device 2.0 (Bruker Daltonics) in 60 consecutive cycles (the sample was rotated 180° after 30 cycles) of 41 seconds (1 s spraying, 10 s incubation time, and 30 s of active drying). The matrix solvent selection was optimised to visualise the spatial distribution of both aerophobin-2 and aeroplysinin-1, which differ in polarity.

Samples were analysed in an UltrafleXtreme MALDI TOF/TOF (Bruker Daltonics), operated in positive reflector mode using flexControl 3.0. The analysis was performed in the 100-1500 Da range and with 30 % laser intensity (laser type 4), accumulating 1000 shots by tanking 50 random shots at every raster position. External calibration of the acquisition method was performed using Peptide Calibration Standard II (Bruker Daltonics) containing

Bradykinin1-7, Angiotensin II, Angiotensin I, Substance P, Bombesin, ACTH clip1-17, ACTH clip18-39, and Somatostatin 28. Spectra were processed with baseline subtraction in flexAnalysis 3.3 and corrected internally using the peaks of HCCA ($[M+H]^+$ *m/z* 190.0499 and $[2M+H]^+$ *m/z* 379.0925). MALDI-imaging MS data was visualised in SCiLS Lab 2015b (SCiLS, Bremen, Germany).

Supplementary Table 1. Number of biological replicates for microscopy and MALDI-imaging MS. C: control; **G**: grazing; **M**: mechanical damage.

	Microscopy			MALDI-imaging MS		
**Experiment**	**C**	**G**	**M**	**C**	**G**	**M**
**3h-2017**	4	4	3			
**1d-2017**	4	4	4	4	4	4
**1d-2016**	4	4	3			
**3d-2016**	4	4				
**6d-2016**	4	4	3			
**Total**		53			12	

Supplementary Table 2. Used raster size, measured area, and number of mass spectra acquired by MALDI-imaging MS from 1-d sponge specimens. Individual: sponge individual; C: control; G: grazing; M: mechanical damage.

Individual	Treatment	Raster [μm^2]	Area [mm^2]	Number of spectra
1	C	250 x 250	131.06	2097
1	G	250 x 250	167.06	2673
1	M	250 x 250	233.44	3735
2	C	275 x 275	131.29	1736
2	G	275 x 275	216.89	2868

2	M	275 x 275	248.20	3282
3	C	275 x 275	199.88	2643
3	G	275 x 275	235.50	3114
3	M	275 x 275	223.09	2950
4	C	300 x 300	266.40	2960
4	G	300 x 300	140.94	1566
4	M	300 x 300	309.60	3440

Supplementary Table 3. Statistical comparison of the spherulous cells density from the surface to the interior. The comparisons were performed for each region of interest (ROI), defined from the surface to the sponge interior with a depth of 100 µm and a length of 500 µm. The values in the table correspond to the p-values of the generalised linear mixed-effects model via penalised quasi-likelihood (glmmPQL) test. Significant values are in bold. Note that there was no mechanical damage group at 3d. Superscript number 1 and 2 denotes experiments performed in 2016 and 2017, respectively.

		ROI 1	ROI 2	ROI 3	ROI 4	ROI 5	ROI 6
**Grazing *vs* Control**	3h[2]	**0.023**	> 0.1	> 0.1	> 0.1	> 0.1	> 0.1
	1d[2]	**0.035**	**0.013**	**<0.001**	**<0.001**	**0.002**	**0.021**
	1d[1]	0.056	> 0.1	> 0.1	**0.041**	**0.001**	**0.025**
	3d[1]	> 0.1	**0.036**	> 0.1	> 0.1	> 0.1	**0.030**
	6d[1]	> 0.1	> 0.1	> 0.1	> 0.1	0.064	> 0.1
**Mechanical-damage *vs* Control**	3h[2]	> 0.1	> 0.1	> 0.1	> 0.1	> 0.1	> 0.1
	1d[2]	**0.013**	**0.009**	**<0.001**	**<0.001**	**0.003**	**0.021**
	1d[1]	**0.022**	> 0.1	> 0.1	**0.036**	**0.001**	**0.011**
	6d[1]	> 0.1	> 0.1	> 0.1	> 0.1	0.080	> 0.1

Supplementary Video 1. Example of *T. perversa* grazing on a sponge in our experimental setup.

Supplementary Video 2. Example of a deterrence experiment showing *T. perversa* choice. *T. perversa* chose a control over a mechanically-damaged sponge piece.

Supplementary Figure 1. Automatic cell counting method. (**a**) Starting at the surface, 6 Regions Of Interest (ROI 1 - ROI 6, yellow squares) with a depth of 100 µm and a length of 500 µm were selected in each microscopic image. Parameters were adjusted to count spherulous cells (blue outline). (**b**) Samples with longer surface area were collected in 2017. To keep each measured area constant with that in 2016, two regions (R1 and R2; each region with an area of 600 µm in depth x 500 µm in length) in each microscopic image were selected by avoiding spongin fibers and aquiferous canals. For each region, the same counting method was applied for 6 ROIs (see **a**). Then, the average density of spherulous cells from these two regions was calculated for each ROI. **c**) Comparison between automatic and manual counting method by counting samples from 2016. Both methods showed a similar pattern of the cell density. Manual counting was done by using "Multi-Point" ImageJ tool.

Supplementary Figure 2. Microscopic section showing shedding and debris in wounded samples collected at 1d. (**a, b**) Shedding: microscopic images at 400x showed shedding of spherulous cells (**arrowhead**) out of wounded surface (s) and into aquiferous canals (ca) in wounded samples (**a**). TEM-image showing shed spherulous cells (**b, arrowhead**) out of wounded surface (s) and an excreted spherule at the surface (**b, arrow**). (**c, d**) Debris in wounded sponges (100x magnification) (**de**) which were excreted into aquiferous canals (**c**) or shed out of wound (**d**). Spongin fibers were also observed to protrude into aquiferous canals or out of the surface (**arrowhead**). ca= aquiferous canal; de= debris; s= wounded surface.

Supplementary Figure 3. Microscopy and photographical recording of recovery after wounding. (**a, b**) Example of grazed sample directly after removal of the sea slug (a). Its osculum (arrowhead) and the edges between the scar and the intact tissue were regenerated after 3d (b). (**c, d, e**) Example of a microscopic section (400x) of samples from control group (c) and from grazing group at 1d (d) and 3d (e). The surface side was at left of each image. Scales at bottom showed the depth from the surface to the sponge interior.

Supplementary Figure 4. Mass Spectra of unidentified brominated compounds from *A. aerophoba* detected by MALDI-imaging MS. Five unidentified Br-containing compounds were detected (possibly as $[M+H]^+$ ions) and listed by *m/z* value (from top to bottom panel). The experimental isotopic pattern (left panel), the potential molecular formula (middle panel), and the theoretical isotopic pattern (right panel) for each compound are showed.

Supplementary Figure 5. MALDI-imaging MS images of ions with an isotopic pattern characteristic of brominated compounds (other than aerophobin-2 and aeroplysinin-1, the first two rows). Relative abundance, experimental most intense *m/z* in the isotopic pattern, and expected number of Br atoms (in round brackets) are shown for each unidentified brominated compound in the three different treatments (C=control, G=grazing, and M=mechanical damage) for each biological replicate (Replicate; i.e., specimens of the same sponge individual). The relative intensity from 0 to 100 % of each compound is depicted in a colour scale with warmer colours representing relatively higher intensity and colder colours lower intensity of each compound. White-dotted line = broken or cut edges.

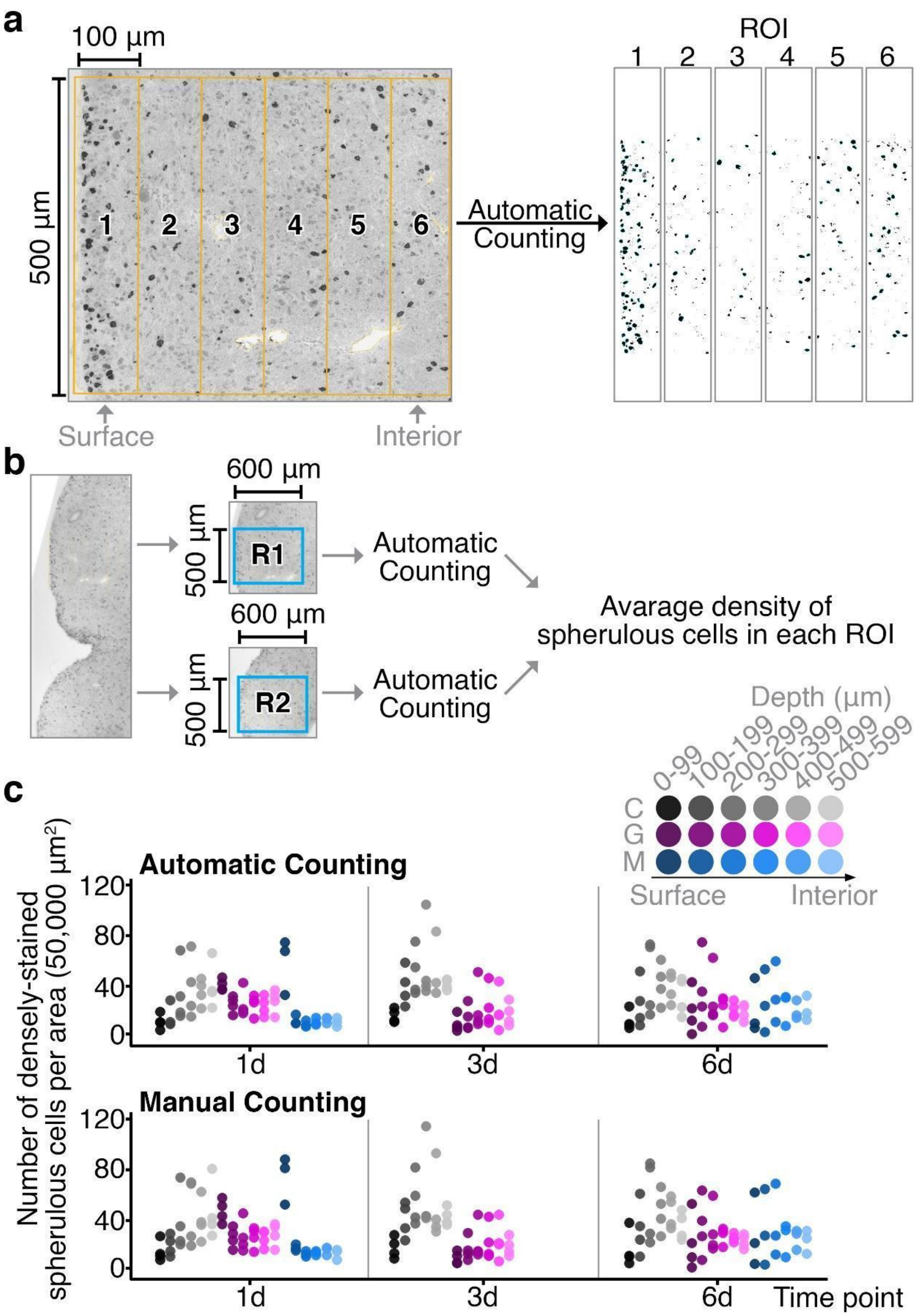

Supplementary Figure 1

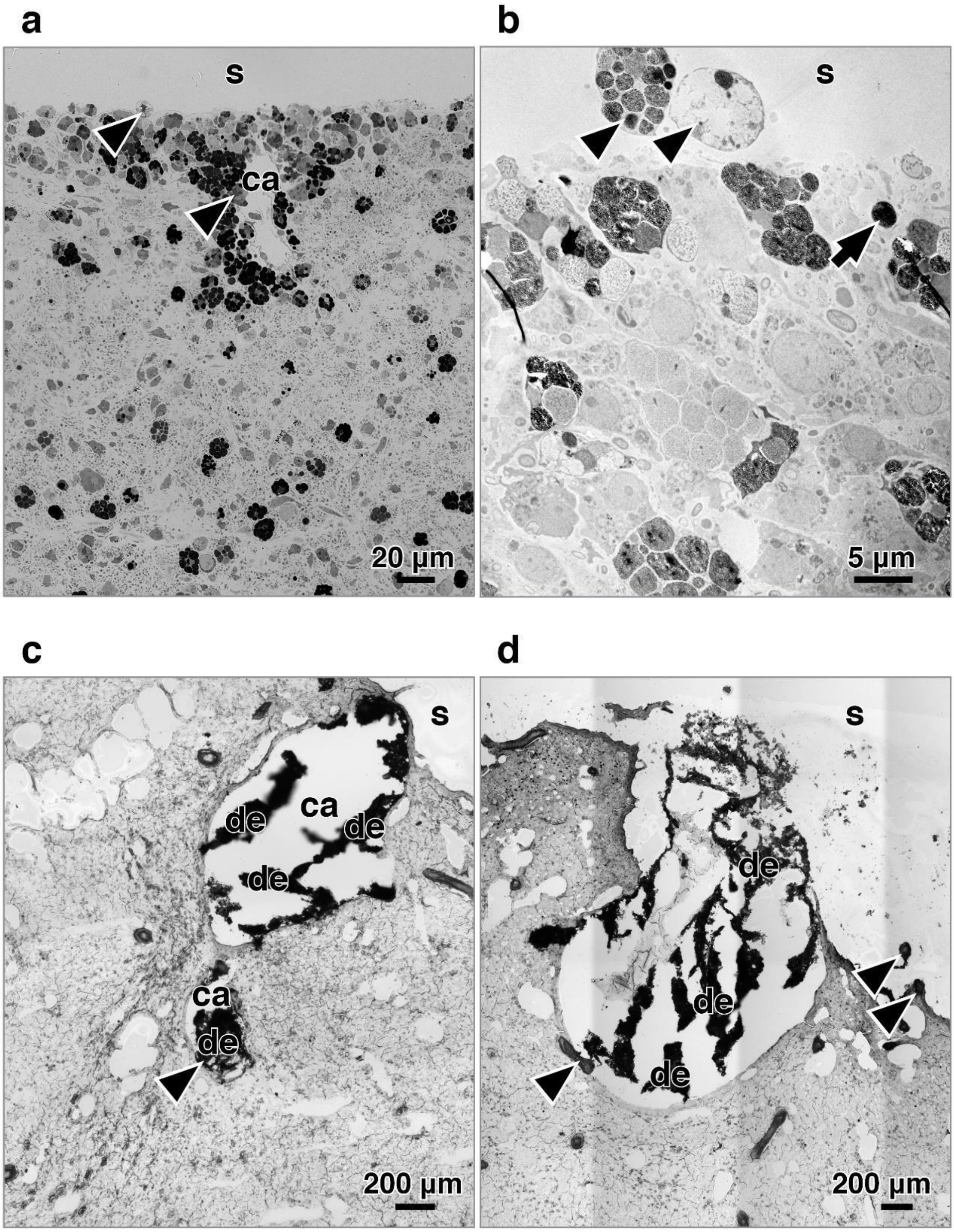

Supplementary Figure 2

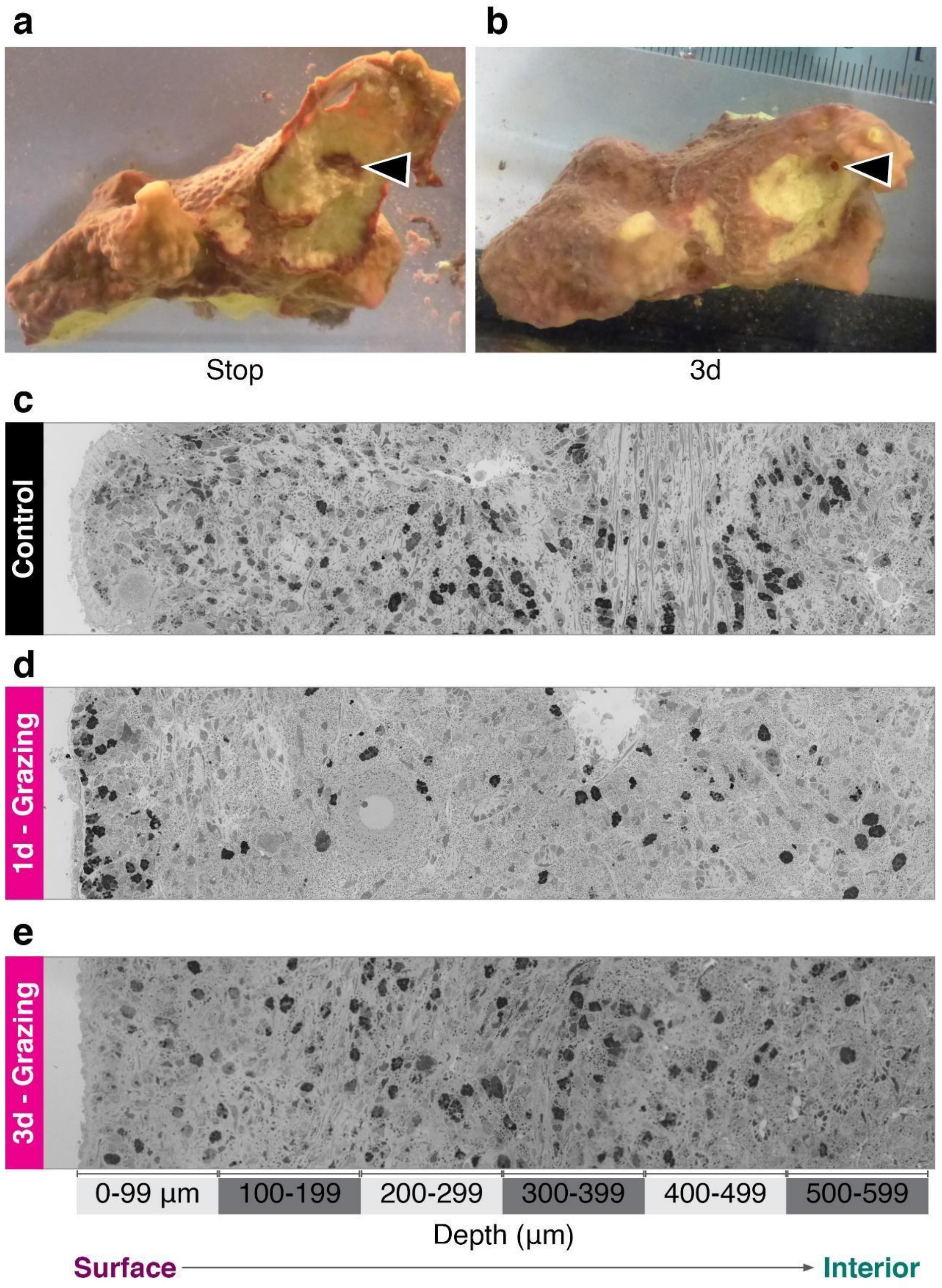

Supplementary Figure 3

143

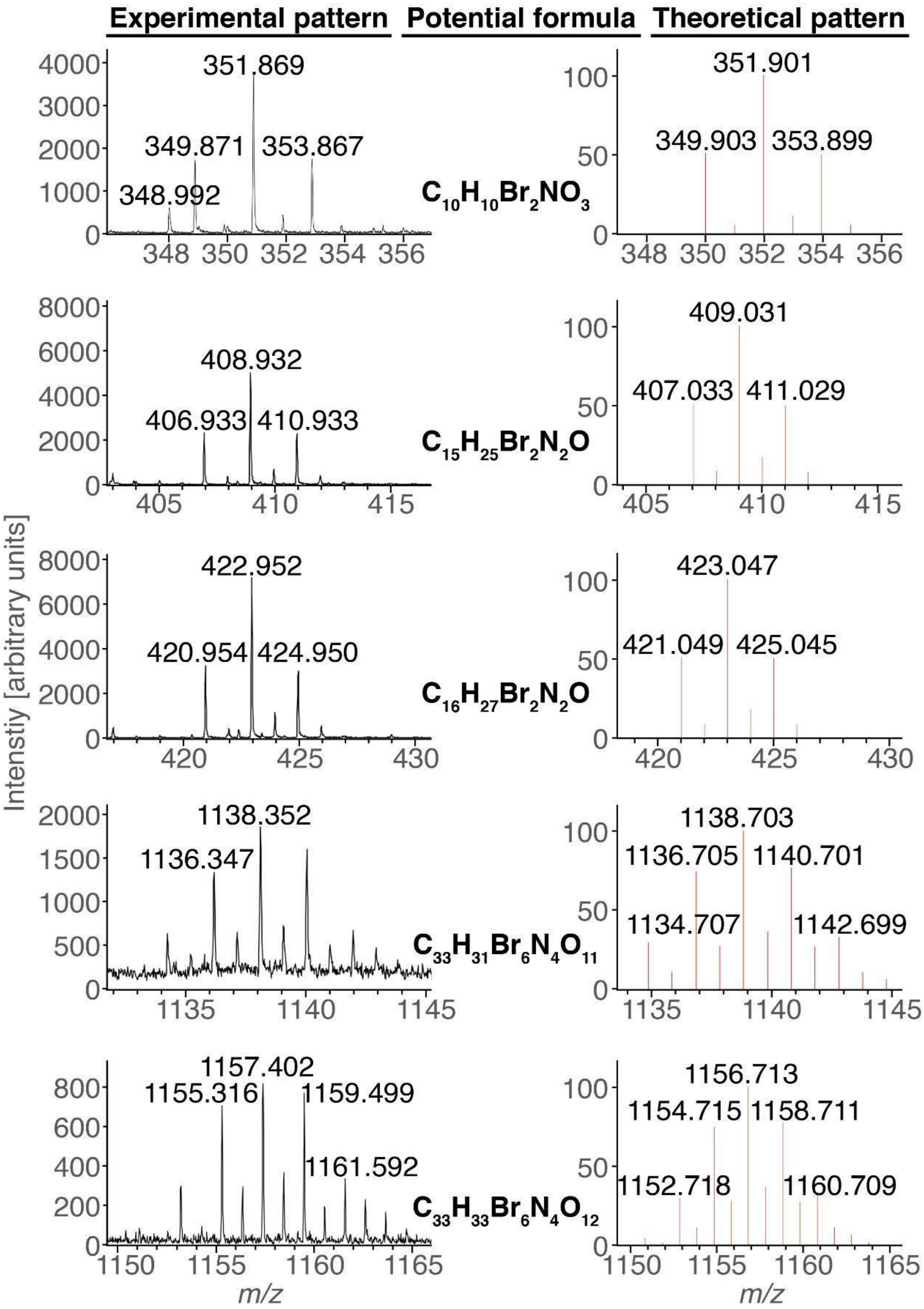

144 Supplementary Figure 4

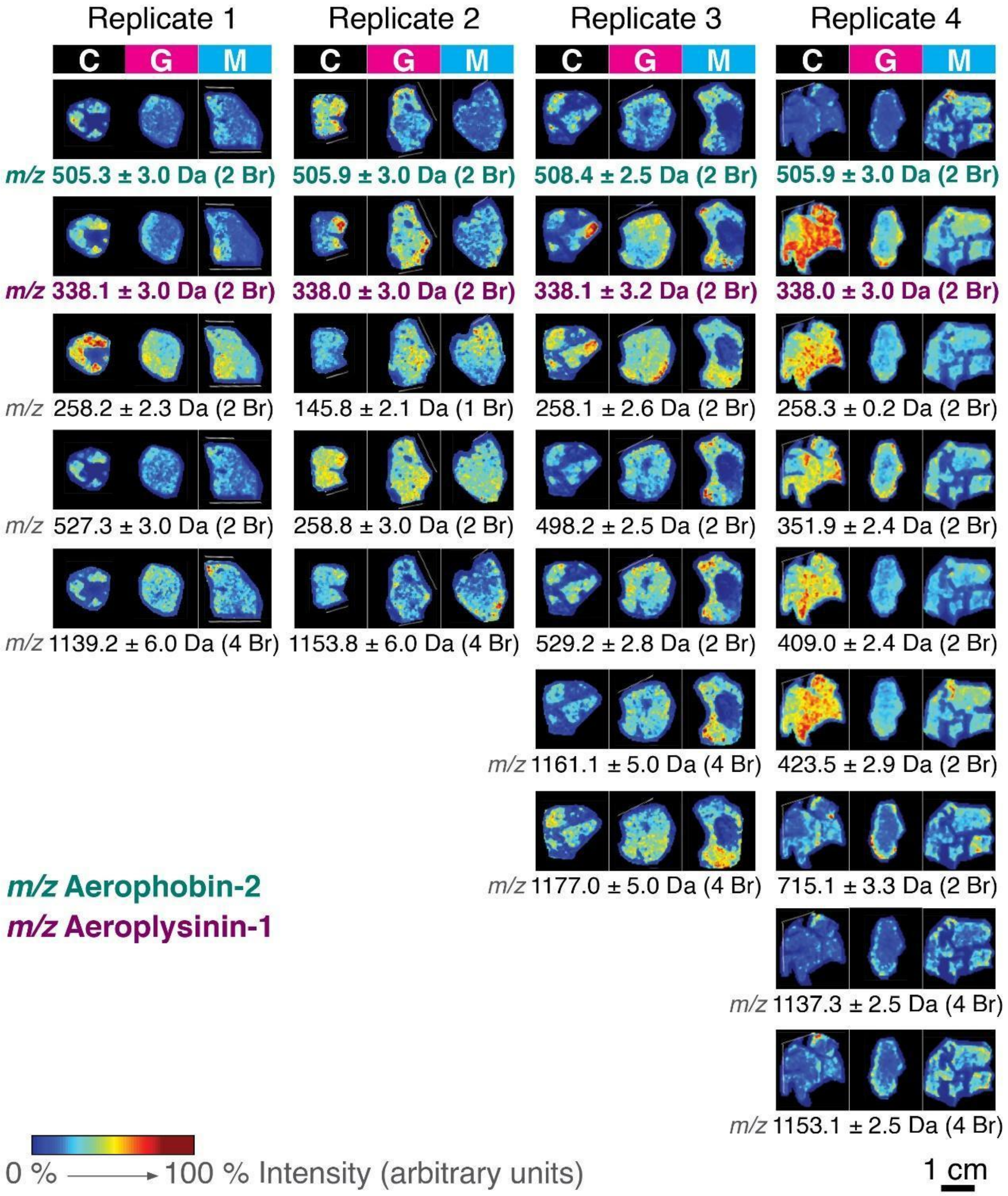

Supplementary Figure 5
